# Surgical Management of Sinonasal Cancers: A Comprehensive Review

**DOI:** 10.3390/cancers13163995

**Published:** 2021-08-08

**Authors:** Florian Chatelet, François Simon, Vincent Bedarida, Nicolas Le Clerc, Homa Adle-Biassette, Philippe Manivet, Philippe Herman, Benjamin Verillaud

**Affiliations:** 1 Otolaryngology—Head and Neck Surgery Department, Hôpital Lariboisière, Assistance Publique—Hôpitaux de Paris, 75010 Paris, France; florian.chatelet@aphp.fr (F.C.); vincent.bedarida@aphp.fr (V.B.); nicolas.leclerc@aphp.fr (N.L.C.); philippe.herman@aphp.fr (P.H.); 2 Department of Paediatric Otolaryngology, Hôpital Necker—Enfants Malades, Assistance Publique—Hôpitaux de Paris, 75015 Paris, France; f.simon@aphp.fr; 3 INSERM U1141 “NeuroDiderot”, Université de Paris, 75010 Paris, France; homa.adle@inserm.fr (H.A.-B.); philippe.manivet@aphp.fr (P.M.); 4 Department of Pathology, Hôpital Lariboisière, Assistance Publique—Hôpitaux de Paris, 75010 Paris, France; 5 APHP, Biobank Lariboisière BB-0033-00064, Platform of BioPathology and Innovative Technologies in Health, Hôpital Lariboisière, 75010 Paris, France

**Keywords:** sinonasal cancer, nasal cavity, paranasal sinuses, treatment, surgery, endoscopy

## Abstract

**Simple Summary:**

Sinonasal cancers are rare. Surgery is often the first step of the therapeutic strategy, possibly complemented by adjuvant therapies. Surgical excision can be performed by an open approach with external incision, by an endoscopic approach using the nasal fossa as a surgical corridor, or by a combination of both. This article describes the main surgical techniques that can be used for the management of sinonasal cancer and reviews the criteria that lead to the choice of one technique over another.

**Abstract:**

Surgery plays an important role in the treatment of sinonasal cancer. Many surgical approaches have been described, including open, endoscopic, or combined approaches. The choice is based on several criteria: general criteria related to the oncological results and morbidity of each technique, specific criteria related to the tumor (tumor extensions, tumor pathology), the patient, or the surgeon himself. The aims of this review are (i) to provide a complete overview of the surgical techniques available for the management of sinonasal malignant tumors, with a special focus on recent developments in the field of transnasal endoscopic surgery; (ii) to summarize the criteria that lead to the choice of one technique over another. In particular, the oncological outcomes, the morbidity of the different techniques, and the specificities of each histologic subtype will be discussed based on a comprehensive literature review.

## 1. Introduction

Sinonasal cancers are rare tumors, corresponding to 3–5% of head and neck cancers, and less than 1% of all cancers [1]. They are characterized by significant histological heterogeneity, with squamous cell carcinoma being the most frequent, followed by adenocarcinoma, melanoma, esthesioneuroblastoma, and adenoid cystic carcinoma [1,2]. Most of these tumors are difficult to diagnose as they are often asymptomatic and are discovered at a locally advanced stage. Surgery plays an important role in the treatment of these tumors and is considered the gold standard of treatment when feasible, possibly complemented by adjuvant therapies. However, the proximity of the tumor’s location to important anatomical structures such as the orbit and the skull base makes the treatment difficult. Over the past two decades, the development of endoscopic sinus surgery has encompassed the management of these cancers. Initially developed for functional sinonasal pathologies, the endoscopic approach was applied to benign pathologies, then to benign tumor pathologies, before being extended to the management of malignant tumors, with the global objective of decreasing surgical morbidity.

The aims of this review are (i) to provide a complete overview of the surgical techniques available for the management of sinonasal malignant tumors, with a special focus on recent developments in the field of endoscopic surgery; (ii) to summarize the criteria that lead to the choice of one technique over another. In particular, the oncological outcomes, the morbidity of the different techniques, and the specificities of each histologic subtype will be discussed based on a comprehensive literature review.

## 2. Which Surgical Techniques Are Available?

### 2.1. External Surgery

Open approaches used to be the only option for the surgical treatment of sinonasal cancers. Since the emergence of endoscopic endonasal surgery, most have been abandoned although an external approach is still sometimes necessary.

#### 2.1.1. Transfacial Approaches

These approaches allow exposure of the midface bone structure and can be used for tumors in all the sinonasal regions. Tumors that have a limited extension to the skull base can also be approached inferiorly. There are two main types of transfacial approaches: the lateral rhinotomy approach and its variations, and the sublabial approach.

**Figure 1:**
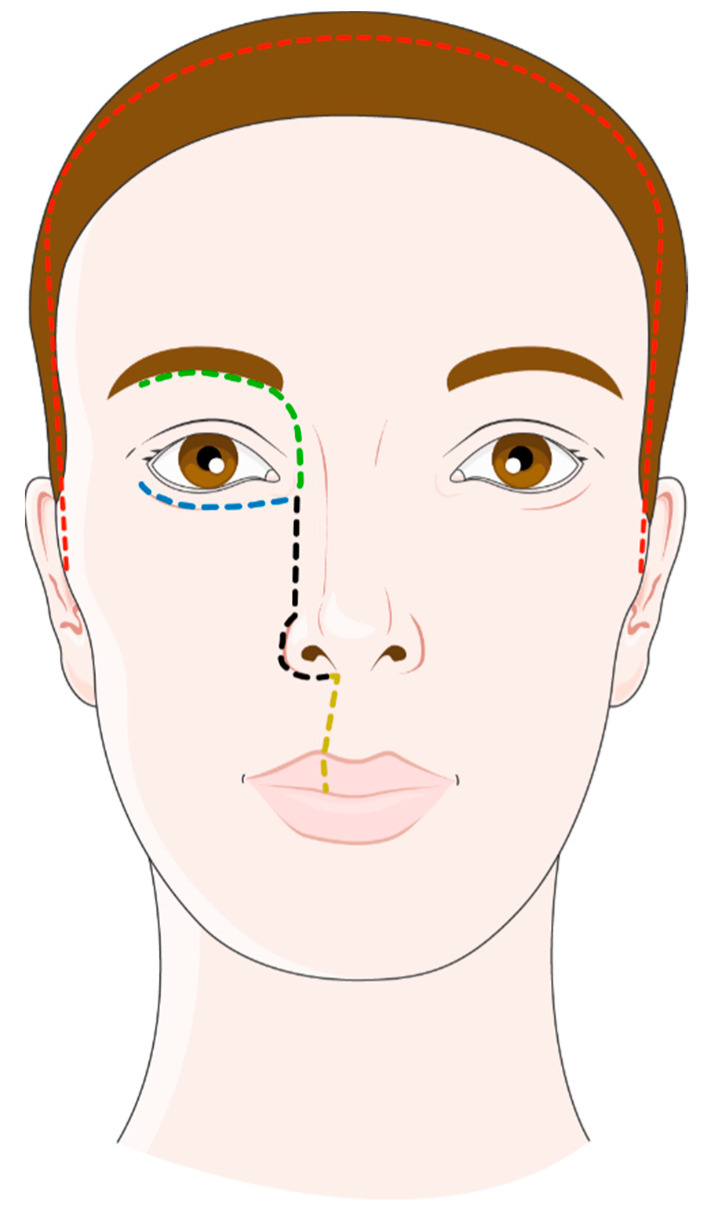
Open approaches for the tumors of the sinonasal cavities: lateral rhinotomy (black dotted line); Weber–Fergusson incision combining lateral rhinotomy with a subciliary split (blue dotted line) and a labial split (yellow dotted line); Lynch incision (green dotted line); and coronal incision (red dotted line). Figure adapted from Servier Medical Art (https://smart.servier.com, accessed on 27 June 2021).

In the lateral rhinotomy, the skin incision begins at the level of the medial canthus and continues into the nasofacial sulcus and the alar-facial sulcus to contour the nasal wing (Figure 1). The soft tissues can be reclined to expose the anterior aspect of the maxillary sinus, the piriform aperture, and the orbital rim. The infraorbital nerve can be either preserved or sacrificed, depending on carcinologic requirements. Extensions of the skin incision can be made to expose the anterior aspect of the maxilla more laterally, up to the maxillary tuberosity and the zygoma: the Lynch incision uses a split under the eyebrow, whereas the Weber-Fergusson incision combines a subciliary split with a labial split (Figure 1). It should be noted that the gain in exposure provided by these different approaches remains relatively unstudied [3]. The lateral rhinotomy approach offers access to the maxilla, ethmoid, sphenoid, and, when associated with a Lynch incision, to the frontal sinus and the anterior aspect of the skull base. The lateral rhinotomy may further be combined with a skin incision along the lower floor of the orbit and osteotomies of the palate, the zygomatic arch, and the maxillary bone below the orbit. Thus, a maxillary swing may be performed giving wide access to the skull base, the infra-temporal fossa, and the carotid artery [4].

In the sublabial approach, the incision is made at the level of the mucosa of the upper vestibule, straight down to the bone, and exposure is accomplished by elevating the periosteum and the soft tissues in the same manner as in the lateral rhinotomy approach. The main interest of the sublabial approaches is, therefore, to allow access to the mid-face skeleton while avoiding a skin incision, but exposure is often more limited than in transfacial approaches. Sublabial approaches performed in an oncologic context are essentially the Rouge Denker and degloving approaches [5].

In the Rouge Denker approach, the superior vestibular mucosal incision exposes the anterior aspect of the maxilla, which is then resected by widening the lateral border of the piriform aperture and resecting the lateral wall of the nose to gain extended access to the maxillary sinus and ethmoid.

The degloving approach begins with a bilateral sublabial incision from one maxillary tuberosity to the other. The periosteum and soft tissues are elevated, up to the orbital rim, preserving the infraorbital nerves. A circular incision is made in the nostril vestibules and the soft tissue can be reclined superiorly exposing the anterior aspect of both maxillae and the nasal skeleton. Depending on the location of the tumor, an antrostomy may be performed in the maxilla, or in some cases a Lefort I osteotomy to expose the tumor [5].

These approaches allow access to the maxillary sinus, the ethmoid sinus, the sphenoid sinus, and the three levels of the skull base. Their indications have strongly decreased in favor of endoscopic approaches.

#### 2.1.2. Craniofacial Resection

In the case of tumors extending to the anterior skull base, this approach, which combines a transfacial and a high transcranial approach in the same surgical procedure, allows the lower intrasinusal part of the tumor and its intracranial component to be accessed. It also allows the resection of a possible limited orbital invasion. This is a validated technique, described and used for more than 40 years [6,7].

The procedure begins with a lateral rhinotomy approach with a resection of the maxillary, ethmoidal, and/or sphenoidal extensions of the tumor. The cribriform plate is exposed from below. A coronal incision is then made (Figure 1), followed by a bifrontal craniotomy with a median craniotomy beginning at the glabella and extending upward 5 cm from one pupillary line to the other. The frontal lobes are reclined, allowing exposure of the intracranial tumor contingent and the floor of the anterior cranial fossa. Depending on the existence of a dural invasion by the tumor, the dissection is done either extradurally or intradurally, removing the dura mater and possibly the invaded brain tissue. The dura is reconstructed using a pericranial flap harvested during the approach.

The frontal craniotomy can be replaced by a subfrontal transcranial approach when there is a very limited low extension of the tumor. A coronal approach allows a naso-orbital-frontal bone flap to be made and the skull base to be exposed from the frontal bone to the dorsum sellae. The dura may be resected as need be, from top to bottom only, thus with a limited visualization of the lower part of the ethmoid and maxillary sinuses [8].

These different approaches can be extended to other regions depending on the tumor extensions. It can be extended to the orbit if exenteration is necessary [9]. The incision includes the invaded eyelids and the periorbital skin in case of invasion requiring exenteration or is subciliary in case of orbital clearance. The dissection allows the removal of all the orbital contents to finally access the orbital apex, which must be ligated before being sectioned to avoid retraction of the ophthalmic artery that may cause intracranial complications [10].

### 2.2. Endoscopic Surgery

Developed over the last thirty years by surgical teams performing functional endoscopic sinus surgery, the endoscopic approach was initially used for the removal of benign sinonasal tumors and has been progressively extended to malignant pathologies of sinonasal location with the development of endoscopic skull base approaches and trans-nasal craniotomy techniques [8,11,12]. Endoscopic sinonasal oncologic surgery is based on a strategy centered on the tumor insertion area, as initially described for inverted papilloma surgery [13], and uses a multi-layer centripetal resection technique that involves several steps [12]:

1. Tumor debulking and identification of the tumor attachment site.
2. Centripetal removal of this tumor attachment site.
3. Removal of at least one additional safety plane in relation to the planes invaded by the tumor: depending on the case, it may be necessary to remove the cribriform plate, the dura, the olfactory bulb, the lamina papyracea, and the periorbita
4. Biopsies from the edges of the resection for frozen section and final histological analysis
5. Reconstruction if necessary, including duraplasty if the dura has been resected.

The essential elements of each of these steps are outlined below.

#### 2.2.1. Installation

The procedure is performed under general anesthesia, with oro-tracheal intubation. The patient is in a supine position with a head-up tilt to limit bleeding. The nasal cavity is packed with a vasoconstrictive agent before starting the procedure. The surgery is performed with a 0° or 30° endoscope and 4 mm in diameter. A sub-mucosal infiltration with epinephrine and xylocaine can be performed to facilitate elevation and limit bleeding. If it is possible carcinologically, the mucosal flaps that will be used for the reconstruction, such as the nasoseptal flap, are elevated at this moment of the surgery and placed at a distance from the operating field to avoid any damage during the procedure.

#### 2.2.2. Identification of the Tumor Attachment Site

**Figure 2:**
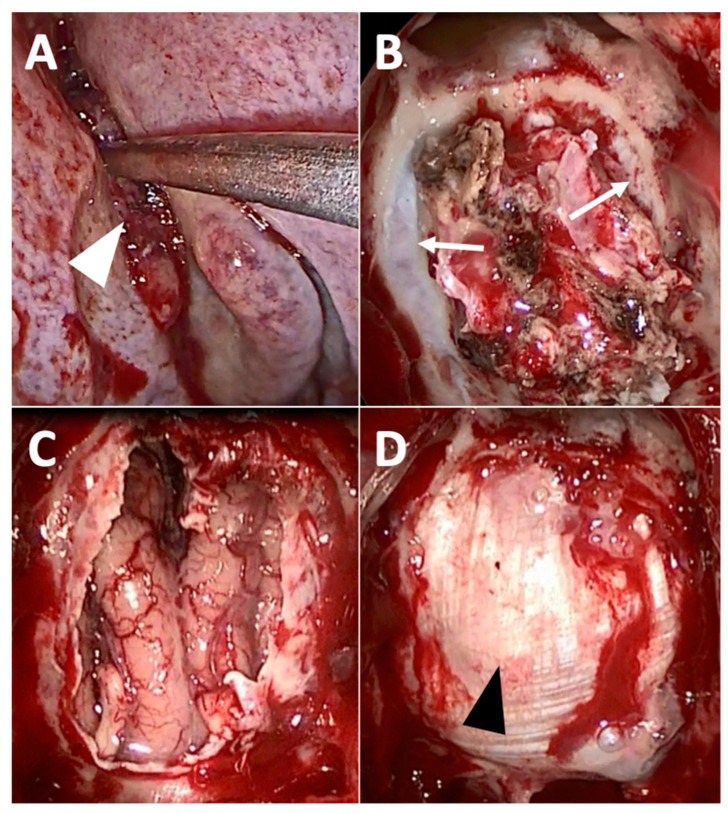
Exclusive endoscopic resection of intestinal-type adenocarcinoma of the left olfactory cleft. (**A**) After debulking, the tumor attachment site (white arrowhead) is exposed within the left olfactory cleft. (**B**) Septectomy, bilateral ethmoidectomy, the opening of the frontal sinuses (Draf 3 procedure) and removal of the nasal part of the tumor has been performed; the skull base has not been removed yet, but the dura mater (white arrow) is exposed from either side of the olfactory clefts. (**C**) Complete removal of the tumor has been performed, with transnasal craniectomy (removal of the cribriform plates, of the roof of the ethmoid, and the dura mater), exposing the frontal lobe. (**D**) Final aspect after duraplasty with fascia lata (black arrowhead).

Surgery most often begins with tumor debulking, using a microdebrider or cold instruments, to delineate and visualize the areas of insertion and invasion of the tumor (Figure 2A). Depending on the location of the tumor and its extensions, certain exposure techniques may be necessary: medial maxillectomy with a frank section of the lacrimal duct, prelacrimal approach, or Sturman Canfield (Denker endoscopic) approach for access to the maxillary sinus and/or infratemporal fossa [14,15,16,17]; the endoscopic opening of the frontal sinus (Draf I, II, or III technique) for extensions to the frontal sinus or the anterior skull base; a septectomy is often associated, allowing 4-handed surgery (Figure 2B).

#### 2.2.3. Tumor Removal

The procedure continues with a “centripetal” tumor removal, starting at the periphery of the tumor attachment zone, respecting a macroscopic margin of healthy tissue, and progressing towards the center of the tumor insertion zone, allowing the removal of the tumor. It should be noted that monobloc resection is rarely possible, but complete resection of the tumor insertion zone remains the most important requirement.

#### 2.2.4. Excision of at Least One Additional Plane in Relation to the Invaded Planes

If the lesion is purely mucosal, the underlying healthy bony plane (septum, skull base, orbital bone) must be resected. In the case of a tumor of the olfactory cleft, the cribriform plate must be resected: both cribriform plates used to be removed endoscopically, but it has been shown recently that a unilateral removal of the cribriform plate was possible, especially in the case of certain small tumors [18,19]. In practice, the craniectomy is done with a burr, with a bone section taking away the cribriform plate, the ethmoid roof, and the crista galli. The ethmoidal arteries are cauterized and sectioned at this point of the surgery to avoid retraction into the orbit and an orbital hematoma. If the lesion has invaded the cribriform plate or crossed the skull base, removal of the dura and the olfactory bulb is necessary (Figure 2C). An extension to the rectus gyrus of the frontal lobe is also possible [20]. Some tumors, notably olfactory neuroblastomas, present frequent intradural extensions and require an almost systematic extended resection of this type. Similarly, tumors that are in contact with the orbit may require resection of the lamina papyracea and/or of the periorbita.

#### 2.2.5. Frozen Section of the Margin Resection

Biopsies with extemporaneous analysis at this stage of the procedure check for clear resection margins. Precise mapping of the resection margins is also useful for the planning of postoperative radiotherapy. Some teams have proposed the use of anatomical diagrams to precisely document the resection margins and facilitate future multidisciplinary discussion of tumor management [20].

#### 2.2.6. Reconstruction

In some cases, no reconstruction is necessary. If a dural resection has been performed, a duraplasty is required. In the case of an extensive defect, a multilayer technique is preferred [21]. The iliotibial tract is most often used (Figure 2D): taken from the patient’s thigh, it is cut into 3 pieces which are positioned in 3 layers to close the defect: an intracranial intradural layer, an intracranial extradural layer, and an extracranial layer placed on the roof of the nasal cavity [22]. The reconstruction can then be covered by a vascularized flap to protect the graft and facilitate healing [23]. Many local flaps (nasoseptal flap, turbinate flap, etc.) or regional flaps (temporal fascia flap, pericranial flap, etc.) have been described. The use of a free flap is sometimes necessary (anterolateral thigh flap, forearm flap, etc.) [24]. The most commonly used is the nasoseptal flap, pedicled on the posterior nasoseptal artery, a branch of the sphenopalatine artery, and whose surface area almost corresponds to that of the entire nasal septum [25]. Regional flaps are used when local flaps are not available due to carcinological reasons or when their pedicle has been compromised during a previous procedure. The temporoparietal fascia flap is pedicled on the superficial temporal artery. It is then passed under the zygomatic arch, through the infratemporal fossa and a window in the posterior wall of the maxillary sinus, harvested from the nasal cavity and positioned to cover the defect [26]. The pericranial flap, elevated after a bicoronal incision, is slid into an opening in the anterior wall of the frontal sinus and deployed via the frontal sinus opening (Draf III) previously made on the basicranial defect [27].

The adjunctive use of lumbar drain after skull base reconstruction is sometimes used to decrease the rate of postoperative CSF leakage. However, its use remains debated [28], even if it could be of interest in the case of high flow leakage [29].

Other defects may require reconstruction. Reinforcement of the medial wall of the orbit with a simple Silastic blade can prevent enophtalmy and diplopia [30]. Large defects of the maxilla most often require the use of free flaps [31], as well as exenteration [32].

### 2.3. Combined Open and Endoscopic Approaches

They can be performed in the case of tumors that are located in both the intracranial and extracranial compartments and for which the intracranial invasion is too important for a purely endoscopic approach [33,34]. It has also been shown that they can be of benefit to treat certain maxillary tumors: when an open maxillectomy has to be performed, the section of the lateral, posterior, and medial walls of the maxillary sinus, as well as the section of the pterygoid process, can be precisely performed during the endoscopic approach [35,36]. An endoscopic contralateral transmaxillary approach has also been developed, using a sublabial approach to introduce the endoscope and reach out even further laterally or to better control the internal carotid artery [37].

### 2.4. Neck Dissection

Neck dissection in sinonasal cancers is usually recommended only if there is clinico-radiological lymph node involvement. The impact of prophylactic neck dissection is still discussed as occult lymph node involvement is rare, but in some situations, prophylactic dissection could decrease the risk of regional recurrence [38]. The extent of prophylactic neck dissection and its exact indications have yet to be defined [39].

## 3. What Are the Decision Criteria?

When surgical treatment is considered for a patient with sinonasal cancer, the surgeon must choose between the different possible approaches, open, endoscopic, or combined. The choice is based on several criteria: general criteria related to the oncological results and morbidity of each technique, specific criteria related to the tumor (tumor extensions, histological type), the patient, or the surgeon himself.

### 3.1. Open Versus Endoscopic Approaches: General Considerations

#### 3.1.1. Oncological Results

The development of endoscopic surgery was accompanied by a questioning of the principles of oncologic surgery that were applied in open surgery. It was generally accepted in oncological surgery that tumor resection should be done en bloc and that tumor fragmentation should be avoided at all costs [40]. However, in the case of sinonasal cancers, the confinement of the tumor and the proximity of many important structures make the en bloc resection complex, and excessive resections of healthy tissue are entailed [41]. It should be noted that this problem was already present in open surgeries [7]. The first tries with endoscopic approaches to sinonasal tumors have demonstrated that, on the contrary, the endoscopic approach allowed better visualization of the limits of the tumor, and thus a more precise and targeted excision. In particular, the endoscopic approach allows optimal visualization of the tumor attachment site, which is poorly identified during the external approaches. Moreover, tumor debulking does not break the oncological principles of complete resection: since the tumor usually grows exophytically in the sinonasal cavities from a limited attachment zone, tumor debulking is performed in a natural cavity, without risking dissemination; it allows to precisely circumscribe the tumor attachment pedicle, which is then ideally resected. A fragmented removal of the attachment zone is also possible: this tumor fragmentation must be planned in an oncological way and allow a complete removal with clear resection margins at the end of the operation [42]. Indeed, it has been shown that the prognosis of patients who underwent initial surgery with incomplete partial resection was worse than that of patients who underwent immediate complete surgery [43]. Piecemeal resection, however, makes histological analysis of the margins, and communication with pathologists, more complex [41]. Interestingly, a similar evolution has been observed in laryngeal cancer surgery, with the conclusion that transoral endoscopic laser laryngeal surgery with fragmented tumor excision was relevant from an oncological point of view [40,41]. Under these conditions, endoscopic surgery does not appear to be associated with a significant difference in terms of surgical margins [44,45,46,47].

The morbidity associated with transfacial resection and the extent of basicranial defects may also delay subsequent treatment. For example, some authors suggest that radiotherapy should be performed 3 months after surgery to allow healing after craniofacial resection [48]. Endoscopic surgery, therefore, reduces this time, reducing the delay to 15 days between diagnosis and radiotherapy compared to craniectomy surgery, in a series of 168 patients matched on the National Cancer DataBase (NCDB) [49]. Different studies comparing the oncological results between the transfacial and endoscopic approaches have shown similar results in terms of survival [46,50,51,52]. For example, the NCDB study by Povolotskiy et al., which included 1595 patients with non-squamous sinonasal carcinoma, found an overall survival of 65.2% in patients treated by endoscopy and 65.4% in patients treated externally (*p* = 0.59) [52]. It should be noted that the analysis of the literature was limited for a long time by the fact that the published studies were retrospective, with, in the first series, tumors of an earlier stage in the group of patients operated by endoscopic approach compared to those operated by external approach. But similar carcinological results between the two surgical strategies have also been found in studies controlling for this retrospective bias [45,47].

#### 3.1.2. Morbidity

Open craniofacial surgery is a major and sometimes disfiguring surgery. It is associated with a high rate of complications. In the multicenter study by Ganly et al. reporting the results of 1193 patients, the postoperative complication rate was 36.3% with 5% perioperative mortality [53]. The main complications were postoperative CSF leaks, pneumencephaly that could be compressive, intracranial infectious complications (meningitis, empyema, etc.), hemorrhagic complications (subdural or intraparenchymal hematoma), and frontal syndrome due to retraction of the frontal lobes. There were also ophthalmological complications, with damage to the optic nerve, oculomotor muscles, or lacrimal tracts. Osteoradionecrosis of the frontal flap has also been described after radiotherapy.

Endoscopic surgery appears to be associated with fewer complications and sequelae. In the series of 239 patients who underwent endoscopic excision reported by Abdelmeguid et al. and of whom 70% were treated purely endoscopically, the complication rate was 29%. These were mainly minor complications (seroma, sinusitis, etc.), 14% were endocranial complications and 6% were CSF leaks [54]. The different studies directly comparing the complication rates between the different approaches did not find any significant difference [46,50,55]. However, it appears that endoscopic surgery is associated with a significantly shorter hospital stay than transfacial surgery, with average lengths of stay of 3–6 days for endoscopic surgery versus 6 to 12 days for transfacial surgery [45,46,47,55,56]. This reduction in hospital stay should be associated with a reduction in costs but has yet to be shown statistically [56]. The endoscopic approach was also associated with a better postoperative quality of life compared to patients operated on with an open approach [57].

The high frequency of CSF leak after endonasal resection of skull base tumors was initially described as a limitation to the use of endoscopy for the resection of these tumors. However, during the last decades, there have been important advances in the reconstruction of skull base defects, which have accompanied the expansion of endoscopic sinonasal surgery. In particular, the use of synthetic absorbable sealants, synthetic dural grafts, fibrin glues, free autografts, and free tissue transfer, have significantly improved postoperative CSF leak rates [58]. The use of multi-layer techniques to close the defects was another important improvement, further decreasing the CSF leak rate [21], which became similar to that of open approaches [59]. For example, endoscopic resection of the anterior skull base results in significant dural defects, which can be efficiently closed using the fascia lata “three layers technique”: the first layer is in an intracranial-intradural position, the second layer in an intracranial-intradural position, and the third layer in a purely extracranial position, upon the roof of the sinonasal cavity [22]. Vascularized flaps can also be used to cover the duraplasty and accelerate the healing process, especially if post-operative radiotherapy is planned. The nasoseptal flap can be harvested easily in most situations, but other flaps may be considered if the tumor invades the septum or the pedicle of the nasoseptal flap [60]. The decrease in the CSF leak rate has reduced the need for lumbar drainage, the low complication rate, the shorter hospital stay and has accelerated the dissemination of the technique [60].

### 3.2. Tumor Extensions

#### 3.2.1. Intracranial Extension

Brain invasion is usually considered as a contraindication to the endoscopic approach as frontal craniectomy could provide better control of surgical margins [8]. However, exclusive endoscopic excision has been reported for tumors with limited brain invasion. Recently, a study reported the results of a purely endoscopic approach in a series of 11 patients with midline tumors, some of which had limited aggressive potential (esthesioneuroblastoma, adenocarcinoma) and some of which had potentially aggressive tumors (neuroendocrine carcinoma) [61]. The rate of positive margins was relatively high (55%) but was due to dura invasion rather than brain invasion for which positive margins were found in only 1 patient [61]. This exclusive endoscopic approach was associated with limited morbidity, with 10.5% of minor complications and no major complications. The purely endoscopic approach thus seems possible in patients with limited brain invasion. The problem remains to define the limited aspect: the authors proposed to use an open approach in case of invasion largely exceeding the rectus gyrus or the medial orbital gyrus, or invasion of the sagittal sinus [61].

#### 3.2.2. Orbital Invasion

Invasion of the orbit is a specific issue in sinonasal tumors. The frequency of this invasion varies from 50 to 80% depending on the tumor location, and it is more frequent in ethmoidal tumors where the invasion of the orbit is found in 62–82% of cases [10]. It is a poor prognostic factor in terms of local control and overall survival [48,62,63,64]. Diagnosis can be difficult: ophthalmic clinical signs are not always associated but may be the result of extrinsic compression, and cases of asymptomatic invasion have also been described [65]. A CT scan can accurately diagnose bone erosion of the lamina papyracea and is still effective in assessing fat invasion, but it is difficult to distinguish periorbital involvement from compression [66]. MRI is a powerful tool for this evaluation and has a superior diagnostic performance to CT for soft tissue evaluation. It can diagnose invasion of the papyraceous lamina, extraconal fat, or ocular muscles, with an accuracy of more than 80%, but remains suboptimal for assessing invasion of the periorbitis and intraconal compartment [67]. Imaging results are even more difficult to interpret in cases of induction chemotherapy or previous surgery, and microscopic invasion or perineural invasion may not be detected [67]. These data underline the importance of intraoperative frozen sections to diagnose orbital invasion with the highest accuracy [10].

To standardize the evaluation of this invasion, several authors have proposed classifications [10,67,68,69]. Ianetti et al. developed a classification of orbital invasions using three grades, grade I corresponding to the invasion of the bony wall of the orbit, grade II to the invasion of the extraconal fat, and grade III to the invasion of the oculomotor muscles, the ocular globe, the apex, or the optic nerve [68]. A variation has been proposed by Turri-Zannoni et al. who suggest separating Ianetti’s grade III into a grade III corresponding to the invasion of the contents of the orbit, i.e., the anterior 2/3 of the orbit, with the ocular extrinsic muscles, optic nerve, ocular bulb, and eyelids, and a grade IV corresponding to the invasion of the orbital apex [69]. These different classifications have shown a clear correlation with survival, regardless of treatment [69,70]. When necessary, surgical removal of the orbit can be performed. This is called orbital exenteration when the entire orbital contents and eyelids are surgically removed, or orbital clearance when the eyelids and the palpebral conjunctiva are preserved [10].

The surgical indications in the case of orbital invasion have evolved significantly over time. An orbital sacrifice has been recommended for a long time in the case of orbital periosteal involvement [71]. Some authors have subsequently proposed a strategy of orbital preservation, arguing that the oncological results are identical in the case of tumors extending beyond the periosteum [48,72,73,74]. Lisan et al. reported a series of 58 patients who underwent an orbital preservation strategy for invasion extending to the extraconal fat. The indications for orbital clearance were an invasion of the extraorbital muscles, the ocular globe, and the orbital apex. The orbit was preserved in 66% of patients, with a 5-years overall survival of 60% and a local control of 74%, similar to patients who had undergone orbital clearance [75]. These orbital preservation strategies remain debated since other studies have demonstrated a survival gain in patients with exenteration in case of orbital invasion [76], and since adjuvant radiotherapy does not effectively replace insufficiently aggressive surgery [77]. However, the lack of precision on the degree of orbital invasion in these studies does not allow us to conclude. More recently, multimodal organ preservation strategies have been developed [78], with the use of induction chemotherapy or concurrent radio-chemotherapy that have increased the orbital preservation rate, which can reach 76% of cases [69]. In fact, a surgical preservation strategy can only be conceived in the case of clear removal surgery. For Ferrari et al. microscopic invasion of the orbital apex did not significantly decrease survival [67], but other studies have found microscopic invasion to be a risk factor for local recurrence [75]. Thus, currently, the invasion of the lamina papyracea and the periorbita are no longer considered as indications for exenteration. Invasion of the extraconal fat and lacrimal sac are discussed indications for exenteration, and a preservation strategy can be attempted on a case-by-case basis. The use of extemporaneous histological examinations in this situation is very important to obtain a clear margin surgery, guaranteeing the oncological result [75]. An orbital preservation strategy with the use of neoadjuvant therapies can increase the orbital preservation rate, by selecting patients who respond to radio-chemotherapy, and by increasing the rate of operable patients [67,69]. Invasion of the orbital apex remains a poor prognostic factor and is associated with very poor survival, regardless of the surgical strategy adopted; more aggressive treatment could therefore be considered in these situations [69].

Histology of the tumor is a relevant factor in the decision to preserve the orbit or not [79]. Perineural invasion should systematically be suspected in adenoid cystic carcinoma and is often underestimated by MRI [67]. The high risk of locoregional recurrence and especially of distant metastasis in mucosal melanoma suggests avoiding a mutilating procedure such as orbital clearance if possible [69,75]. For some people, a strategy of orbital preservation could be proposed quite widely in the case of olfactory neuroblastoma, as radiotherapy alone could be a valid option from an oncological point of view in case of indication for exenteration [80].

In case of orbital preservation, enophthalmy, diplopia, ectropion, epiphora, canthal dystopia, or decreased visual acuity may alter the functional prognosis. In the series by Imola et al., 37% of patients with orbital preservation had a functional impairment and 9% had a nonfunctional eye. The main cause was malposition of the globe, due to insufficient reconstruction of the orbital rim. Appropriate reconstruction could decrease the risk of having a nonfunctional orbit, using free flaps if necessary [81]. However, even after surgery preserving the orbital contents, there remains a nonfunctional orbit rate of 2–5% with functional impairment in 33–39% [69,75,81]. When exenteration is necessary, reconstruction should be performed at the same time, if possible using a free flap, with better results with a prolonged operating time [10]. The patient should also have systematic psychological support, because of the important impact on the quality of life [32].

#### 3.2.3. Other Tumor Invasions

Apart from brain and orbit invasion, and even if there are very few survival studies specifically on the prognostic importance of these tumor extensions, most authors currently agree to contraindicate exclusive endoscopic management in cases of invasion of the maxillary infrastructure, the anterior wall of the maxillary sinus, orbital floor, the ascending branch of the maxilla, the nasal bones, the anterior or posterior table of the frontal sinus, the soft palate, massive invasion of the pterygopalatine fossa, the infratemporal fossa, invasion of the cranial nerves beyond their foramen in the skull base, invasion of the cavernous sinus or invasion of the soft tissues of the face [39,82]. However, advances in endoscopic techniques allow us today to reconsider these contraindications in certain selected situations and in experienced teams [61,67,69,83].

### 3.3. Histological Type of the Tumor

#### 3.3.1. Squamous Cell Carcinoma

Squamous cell carcinoma is the most common histology, accounting for 42–61% of sinonasal tumors [1,2,76,84]. These tumors are mainly found in the nasal cavity and the maxillary sinus, followed by the ethmoid sinus, and more rarely the frontal and sphenoidal sinuses [1,47]. With a 5-year overall survival of 50–60% [76,85,86], sinonasal squamous cell carcinoma is a poor prognosis tumor. Given its location and aggressiveness, aggressive surgery is often necessary such as an open maxillectomy approach with orbital clearance and free flap reconstruction, depending on the case. The combined approaches with endoscopic selective drilling of the pterygomaxillary junction and medial and posterior osteotomies can be interesting in this situation [35]. Endoscopic surgery remains useful in certain situations. During the 2010–2014 period, 24% of patients with sinonasal squamous cell carcinoma registered in the NCDB had undergone endoscopic surgery and had the same overall survival as those who underwent open surgery [47]. Clear resection margins are an important prognostic factor in squamous cell carcinoma [42] and endoscopic surgery in selected patients does not appear to be associated with a decreased rate of clear margins [42,47]. However, the number of patients managed has been shown to have an influence on the rate of clear margins [42] and the endoscopic approach must therefore be carried out by trained teams.

#### 3.3.2. Adenocarcinoma

Adenocarcinomas account for 7–12% of all sinonasal cancers [1,2,76,84]. They are divided into two histological types, with intestinal-type adenocarcinomas (ITAC) and non-intestinal-type adenocarcinomas (NITAC). They have a higher incidence in Europe than in North America [87]. Sinonasal adenocarcinomas are associated with better survival rates than squamous cell carcinomas [86], with a 5-year overall survival of 54–69% [86,88,89,90,91,92]. ITACs are frequently found in wood or leather workers [90], they develop mainly in the olfactory cleft [93]. The gold standard treatment for ITAC is surgery with clear margins, which may be followed by radiotherapy in case of poor prognosis [94,95]. This is a perfectly standardized procedure and consists of a centripetal resection of the olfactory cleft, associated with a craniectomy and dural resection depending on the tumor extension [11,87]. This surgery can be performed by transnasal endoscopic approach, in an increasing number of indications. However, external or combined approaches are still indicated in case of invasion of the supraorbital dura, the nasal bones, or the soft tissues of the face [80]. A massive invasion of the brain, the orbit, or the lacrimal system are still indications to be discussed on a case-by-case basis, as we have seen previously. Endoscopic resection with transnasal craniectomy has been demonstrated to have similar or even better oncologic results than external craniectomy with reduced morbidity [96,97]. In case of tumor not extending to the midline, unilateral endoscopic resection with transnasal craniectomy can be undertaken, which reduces operative morbidity (with preservation of subjective olfaction in 45% of patients) and shortens the hospital stay [18].

The management of NITACs is less investigated. Although this term covers a heterogeneous group of tumors, they seem to have a similar aggressiveness to ITACs, although this remains debated [89]. As for ITACs, surgery remains the gold standard of treatment [98], completed by adjuvant radiotherapy in case of high-grade tumors [91].

#### 3.3.3. Adenoid Cystic Carcinoma

Adenoid cystic carcinomas are minor salivary gland tumors, representing 5–7% of sinonasal cancers [2,84,99]. They predominate in the maxillary sinus and are characterized by an important propensity to perineural invasion, which means that they are regularly discovered with an extension to the orbit or the skull base, despite a slow evolution [100]. The 5-year overall survival is 63–86%, but 30–35% of patients have a recurrence, and the risk of distant metastasis is important. As a result, the 10-year overall survival of these patients continues to decline to 32–67% [99,100,101,102]. Surgery remains the gold standard of treatment when possible, and obtaining microscopically clear resection margins is associated with a significant improvement in overall survival [99,102]. When the extent of the tumor makes the morbidity of the procedure too high, a subtotal resection is possible, associated with adjuvant radiotherapy [103]. Interestingly, obtaining microscopically positive margins did not show superiority in terms of local recurrence compared to macroscopically positive margins in a retrospective study [104]. Invasion of the cavernous sinus does not necessarily contraindicate surgery, but extensions to the base of the skull and the infratemporal fossa remain factors of poor prognosis [105].

A retrospective study comparing patients with sinonasal adenoid cystic carcinoma treated by open surgery or by endoscopic surgery did not find a significant difference in survival rate, but the small number of patients treated by an endoscopic approach did not allow a definitive conclusion [106]. Two series report the results of 30 and 34 patients treated endoscopically. They report overall survival rates at 5 years of 62.7% and 86.5% and confirm that endoscopy is a valid surgical treatment modality [99,104]. Surprisingly, the study by Kashwazaki et al., which also included patients with adenoid cystic carcinoma of the nasopharynx, reported a clear surgical margin rate of 7% [104] which may seem too low to recommend this surgical strategy. However, the study by Volpi et al. which reported a clear margin rate of 79.4% is in favor of the endoscopic approach with satisfactory carcinological results [99].

#### 3.3.4. Olfactory Neuroblastoma

Olfactory neuroblastoma, or esthesioneuroblastoma, is a rare tumor representing 2–6% of sinonasal cancers [107]. It develops in the olfactory cleft. It is a tumor with a relatively good prognosis, with a 5-year survival rate of 62–83%, but the evolution is variable and some tumors may present an aggressive behavior leading to rapid distant metastasis [108]. A classification was developed by Kadish and is now the most commonly used. Kadish A corresponds to tumors limited to the nasal cavity, Kadish B corresponds to tumors extending to the paranasal sinuses, and Kadish C corresponds to tumors extending beyond the sinonasal complex [109]. Morita et al. secondarily proposed a fourth stage, Kadish D, corresponding to tumors with locoregional or distant metastasis [110]. Other classifications have been developed, and in particular, the Hyams system, a histopathological grading system, found to be an independent predictor of locoregional and overall survival [111] and even potentially the most relevant prognostic factor [112]. Treatment is multimodal, including surgery which is currently considered the mainstay of treatment for olfactory neuroblastoma. In high-grade olfactory neuroblastoma (Hyams 3–4), preliminary results suggest that induction chemotherapy could be considered, followed by chemo-radiotherapy in case of good response [113].

Many studies have investigated endoscopic surgical techniques for olfactory neuroblastoma management, and have found similar or better results, in terms of overall survival and clear margin rate [114,115]. Endoscopic surgery was also associated with fewer intracranial or general complications than craniofacial surgery [114]. In the case of a well-lateralized tumor, unilateral craniectomy has been shown to be associated with good carcinological control, and with preservation of olfaction in 43% of patients [19]. It also enables the harvest of contralateral nasal cavity flaps for reconstruction, such as a septal flap or a flip-flap that can be used for duraplasty [108].

Tumors classified as Kadish A or B should therefore be resected preferentially endoscopically, but this approach can also be used for Kadish C or D tumors. The limits of the endoscopic approach remain the invasion of the maxillary infrastructure, the anterior skull, and facial structures, the orbital floor laterally to the infraorbital nerve, the intraconical orbital compartment, the invasion of the dura laterally to the orbit, or a massive invasion of the brain [116]. An external approach can then be combined to complete the resections. In the case of orbital invasion, a retrospective study suggests that primary radiotherapy with orbital preservation may achieve similar results to exenteration followed by radiotherapy [80], thus an orbital preservation strategy could be undertaken.

#### 3.3.5. Mucosal Melanoma

Mucosal melanomas represent 4 to 8% of sinonasal malignancies [2,117]. They are most often located on the lateral nasal wall, followed by the septum and the maxillary sinus [118]. It is one of the sinonasal tumors with the poorest prognosis, with an estimated 5-year overall survival of 20–35% [118,119], and with a frequent evolution towards distant metastases even in the case of a locally resectable tumor [120]. Surgical removal, when possible, is the gold standard, and is frequently combined with postoperative radiotherapy or less often with chemotherapy [121,122]. Cervical neck dissection is more debated [123]. The recent use of immunotherapy in the treatment of melanoma has been extended to the treatment of mucosal melanoma, although no survival benefit has been demonstrated to date [124]. Endoscopic surgery for the resection of sinonasal mucosal melanomas has been shown to be effective and is associated with similar oncologic outcomes compared to more invasive surgery [125,126,127]. Some studies have found better overall survival [119,128,129], and improved local control [128,130] in patients treated endoscopically compared to open craniofacial surgery, but these results remain controversial and may reflect a selection bias or may be related to the lower morbidity associated with this surgery [119]. Resection with clear resection margins does not provide a survival benefit in mucosal melanoma [117]. The very high risk of early metastatic recurrence despite surgery with clear margins on the primitive lesion suggests that potentially debilitating procedures such as exenteration, amputation of the nasal pyramid, or total maxillectomy should be limited [120]. These indications must therefore be discussed on a case-by-case basis, according to the context, the progression of the disease, and the patient’s wishes. Endoscopic procedures, which are less morbid, can be proposed from the start when they allow complete removal.

#### 3.3.6. Sinonasal Undifferentiated Carcinoma (SNUC)

Sinonasal undifferentiated carcinoma (SNUC) is a rare tumor accounting for 3% of sinonasal cancers [2]. These tumors have been reported to be aggressive, with a 5-year overall survival of 35% according to a study by Issa et al. of 243 patients registered in the NCDB [131]. Moreover, these tumors are discovered in most cases at a locally advanced stage [131,132]. There is no consensus on the best therapeutic strategy, but for some, a multimodal approach with induction chemotherapy, surgery, and then concurrent radiochemotherapy would be associated with better survival [132]. The current trend is to propose chemotherapy as first-line treatment, followed in case of good response by radiochemotherapy, or by surgery in case of failure [111,112].

Endoscopic surgery is possible and 43% of the patients operated on in the series of De Bonnecaze et al. were treated endoscopically [132]. A series of 13 patients with SNUC operated endoscopically reports satisfactory oncological results, without operative or postoperative complications [133], arguing for endoscopic treatment when feasible.

#### 3.3.7. Sinonasal Neuroendocrine Carcinoma (SNEC)

Sinonasal neuroendocrine carcinoma (SNEC) are even more uncommon cancers and are therefore poorly studied. They seem to be associated with a better prognosis than SNUC, with a 5-year overall survival of 70% [134]. Multimodal treatment also seems to be associated with a better prognosis and is currently recommended for the management of these tumors, with surgery remaining the mainstay of treatment, which should be performed in patients with resectable tumors.

#### 3.3.8. Soft Tissue Sarcoma

Soft tissue sarcomas are rare tumors of mesenchymal origin, which can reach the sinonasal tract. The most frequent histology is alveolar rhabdomyosarcoma. It is an aggressive tumor, and the 5-year overall survival is estimated at 31–62% [135,136,137]. The management of these tumors is multimodal. Tumor resection with wide margins, usually recommended in other localizations, is frequently not achievable because of the anatomical proximity of the brain and the orbit [135,136]. A retrospective study found that surgery is a predictive factor of complete response in sinonasal sarcoma, regardless of the resection margin quality, and it should always be performed when feasible [136], however, the recommended wide resection is rarely compatible with endoscopic surgery.

Biphenotypic sinonasal sarcoma is a rare subtype of sinonasal sarcoma recently described [138], presenting as a slow-growing tumor that is usually suitable for surgical resection. The endoscopic management of these rare tumors has been reported [139], but the rarity of this pathology does not allow us to compare the different therapeutic strategies.

### 3.4. Patient-Related Criteria

The patient’s general condition must also be considered to define the most suitable surgical approach. The endoscopic approach is in fact associated with simpler and shorter postoperative courses than with the external approach [45,46,47,55,56] and is, therefore, more adapted to the most morbid patients. In all cases, the modalities and risks of the procedure (open or endoscopic) must be discussed with the patient to establish the surgical strategy. This is particularly important in the case of mutilating procedures such as orbital clearance, which are rejected by some patients.

### 3.5. Surgeon-Related Criteria

The surgical treatment of sinonasal cancer is extremely complex due to the proximity of noble organs. With the emergence of endoscopic surgery, the treatment of these cancers requires an additional technicality, which due to the rarity of these lesions, can only be acquired in specialized centers. For example, studies performed on the NCDB database have shown that the survival of patients with squamous cell carcinoma of the nasal cavity is increased in the center with a large track record of this type of disease [140] and that centers treating more than two patients per year were associated with a higher rate of clear margins [42]. It has been shown that surgical experience may be associated with increased overall survival and local control in patients treated endoscopically for sinonasal adenocarcinoma [92,141]. Similarly, several retrospective studies have shown a decrease in CSF leak rates after endoscopic skull base reconstruction with increased surgeon experience [142,143]. Finally, the equipment available to the surgeon can be a limitation to the endoscopic approach, since the resection of sinonasal tumors requires specific equipment: doppler probe to help identify the internal carotid artery especially in the parapharyngeal space [144], high-resolution optical system, dedicated instruments, drills with burrs and long handpieces...

The experience of the team is therefore important in the choice of the surgical approach, which must be the one that combines the highest rate of clear resection margins with the least operative morbidity.

## 4. Future Directions in the Surgical Management of Sinonasal Cancers

During the 2010–2015 period in a study performed using the American NCDB database, 28% of sinonasal tumors were managed by an exclusive endoscopic approach [84]: this rate is likely to continue to increase in the future as endoscopic techniques and skills are improving. Previously described contraindications to endoscopic surgery, such as invasion of the orbit or brain parenchyma [39,82], are now being challenged with the development of technologies and techniques, and other contraindications should continue to be challenged [61,67,69,83].

New technologies may also help the surgeon to obtain clear surgical margins: for example, fluorescence-guided surgery could better show the limits of tissue invasion and thus help to define the surgical margins [145].

More globally, the whole management strategy is also evolving in the field of sinonasal cancers. The improvements in molecular characterization of these rare cancers help to better classify tumors [146]. The concept of “histology-driven strategy” [147] may even evolve to truly personalized medicine [148]. In this context, the place of surgery in the therapeutic strategy may change in the future.

## 5. Conclusions

In conclusion, surgery is the key treatment in sinonasal cancers management strategy. The development of endoscopic surgery, whose indications are gradually increasing, seems to be associated with equivalent carcinological results and significantly reduced morbidity. However, this procedure requires selected cases and trained teams.

## Data Availability

No new data were created or analyzed in this study. Data sharing does not apply to this article.
